# Depression and its associated factors among adult hypertensive patients attending follow-up in South Gondar zone public hospitals, Ethiopia, 2023

**DOI:** 10.1186/s12888-024-05807-y

**Published:** 2024-05-14

**Authors:** Moges Wubneh Abate, Adane Birhanu Nigat, Agimasie Tigabu, Berihun Bantie, Chalie Marew Tiruneh, Tigabu Desie Emiru, Nigusie Selomon Tibebu, Getasew Legas, Amsalu Belete, Belete Gelaw Walle, Mulualem Gete Feleke

**Affiliations:** 1 Department of Adult Health Nursing, Debre Tabor University, P.O.Box:272, Debre Tabor, Ethiopia; 2 Department of Pediatrics and Child Health Nursing, College of Health Sciences, Debre Tabor University, P.O.Box:272, Debre Tabor, Ethiopia; 3 Department of Psychiatry, College of Health Sciences, Debre Tabor University, P.O.Box:272, Debre Tabor, Ethiopia; 4 Department of Pediatrics and Child Health Nursing, College of Health Sciences, Wolayta Sodo University, Wolaita, Ethiopia; 5 Department of Adult Health Nursing, College of Health Sciences, Wolayta Sodo University, Wolaita, Ethiopia

**Keywords:** Magnitude, Depression, Hypertension, Ethiopia

## Abstract

**Introduction:**

Depression is the most common public health issue affecting the world's population. Like patients with other chronic medical diseases, hypertensive patients experience many intense emotions which increase their risk for the development of depression. This study aimed to assess the magnitude of depression and its associated factors among hypertensive patients in South Gondar zone governmental hospitals, Northwest Ethiopia, 2023.

**Methods:**

An institutional-based cross-sectional study was used in government hospitals of South Gondar Zone. A total of 311 patients were sampled randomly and included in the study. Statistical Package for Social Sciences (SPSS) version 25 was used for analysis. Data were analyzed mainly by using descriptive statistics and binary logistics regression.

**Results:**

A total of 311 patients participated with a 100% response rate. Almost half of the participants were female. The mean age of the respondents was 58.85 years. More than 60% of the respondents had a co-morbid illness. Among participants, 83 (26.7%) of hypertensive patients had depression. Being female, age, uneducated, having poor social support, the presence of co-morbid illness and complications, uncontrolled hypertension, having less than or equal to two dietary regimen and duration of hypertension greater than ten years were significantly associated with depression.

**Conclusion:**

The magnitude of depression was found to be high. This indicated that depression is a common co-morbid illness among hypertensive patients. Healthcare professionals and other stakeholders should consider and diagnose co-morbid diseases like depression among hypertensive patients. It is also better to give particular emphasis to highly vulnerable groups like females, elders, uneducated and those who have poor social support.

## Introduction

Depression is a common mental illness and hypertension is a chronic disease and because of negligence as well as lack of awareness during diagnosis and treatment; depression is co-morbid disease with hypertension [1, 2].

The World Health Organization (WHO), International Classification for Diseases and Related Disorders (ICD-10) describes the criteria for a depressive episode, where at least four items, such as unhappiness/sense of empty/depressed mood, exhaustion or energy loss, loss of interest in activities, lack of emotional reactions, sleep disturbance, motor retardation, loss of appetite, weight loss, and loss of libido are present for two weeks [3].

Depression affects 350 million people around the world, and it will become the leading cause of disability worldwide by the year 2030 [4]. People with chronic diseases are more likely to have depression than those without any physical illness [5]. It is well known that both hypertension and depression emerge from a complex interaction of social, biological, and behavioral factors [6].

Patients with hypertension represent a particularly vulnerable population as they are at higher risk of developing depression [7]. The global burden of depression along with co-morbid hypertension poses a substantial public health challenge, both at the social and economic levels [8]. The effect of depression on patients with hypertension may have a major bearing on daily living activities, quality of life, and healthcare utilization [9]. Untreated co-morbid depression typically leads to a negative impact on the patient´s mental health, which may affect their compliance with the hypertensive treatment and, in some cases, may lead to suicide attempts [10]. Discovering depression earlier make it easier for people to cope with their condition, leading to better health and quality of life [11].

Currently, there is no evidence regarding the magnitude of depression and its associated factors in the study setting. So, the main aim of this study was to assess the magnitude of depression and its associated factors among hypertensive patients on follow-up, Northwest Ethiopia. It will be used as input for policymakers and responsible bodies like the federal ministry of health (FMOH), regional health bureaus, clinicians and scientific community as baseline evidence to give special focus to assess and manage depression among hypertensive patients.

### Research questions (Hypothesis)

1. There is no associations between depression and hypertension or Patients with hypertension will not develop depression (Null hypothesis)
2. There is associations between depression and hypertension or Patients with hypertension will develop depression (Alternative hypothesis)
3. There is no associations between socio-demographic characteristics**,** behavioral factors, and disease and medication related factors with depression among hypertensive patients (Null hypothesis)
4. There is associations between socio-demographic characteristics**,** behavioral factors, and disease and medication related factors with depression among hypertensive patients (Alternative hypothesis)

## Methods

### Study area, design and period

South Gondar zone is found in Amhara Regional State's which located in the Northernwest part of Ethiopia. More than 2.3 million people are served by these hospitals. In this zone there are seven primary hospitals (Addiszemen, Ebinat, Nefasmewucha, Doctor Ambachew memorial hospital, Muja, Mekane-eyesus, and Andabet) and one comprehensive specialized hospital (Debre Tabor comprehensive specialized hospital). Among these hospitals, three hospitals (Debre Tabor comprehensive specialized hospital, Mekane-eyesus and Addiszemen) were selected by using lottery method. An institutional-based cross-sectional study design was conducted in South Gondar zone governmental hospitals among hypertensive patients on follow-up from April 1to May 30/ 2023.

### Source population, study population, inclusion and exclusion criteria

The source populations were all hypertensive patients on follow up in South Gondar zone governmental hospitals. Hypertensive patients in selected governmental hospitals of the South Gondar zone were the study populations. Hypertensive patients on follow up and whose age ≥ 18 years were included in the study and those patients on follow-up having a hearing impairment, unable to communicate and seriously ill were excluded.

### Sample size determination, sampling procedure, technique, and data collection tool

By taking the prevalence of depression 24.19% [12]. Epi-Info version 7 0.2.2.6 was used to calculate the sample by considered confidence interval 95%, power 80% with design effect 1. Based on this the sample size was 282. By adding a 10% non-response rate, the final sample size was 311. A simple random sampling technique using the lottery method was used to select governmental hospitals in South Gondar zone, and proportional allocation of sample based on the number of hypertensive patients follow-up per month was given for each selected hospitals. So based on this the number of hypertensive patients on follow-up at Debre Tabor comprehensive specialized hospital on January, February and March were 440, 415, and 426 respectively. The average of the three months was 427. Three months follow-up in Mekane-eyesus hospital on January, February and March were 290, 281, and 274 respectively. The average of the three months was 282 and the three months follow-up in Addiszemen hospital on January, February and March were 301, 294, and 290 respectively. The average of the three months was 295. Based on this figure proportional allocation of sample was given for each selected hospitals. For Debre Tabor comprehensive specialized hospital 132, Mekane-eyesus hospital 87, Addiszemen hospital 92. Finally, a systematic random sampling technique was used with the interval of 3 at each hospital and the first participants were selected by lottery method. The questionnaire to assess the factors is adapted after a review of different works of literature [1, 10, 12]; while questionnaire to assess the magnitude of depression is adopted from Patient Health Questions 9 (PHQ9) [13]. The questionnaire was prepared in the English version and translated to the Amharic version by a language expert to maintain its consistency. The questionnaire has contained four sections. The first part includes the demographic part contains 7 questions, the second part is concerned with behavioral factors which contain 5 questions, the third part concerned disease- medications related contains 6 questions and the fourth part is about the magnitude of depression questions which contain 9 questions. Social support was assessed by using three-item Oslo social support scale with a range of between 3 and 14. Based on this the score of 12 to 14 was classified as strong social support, the score of 9–11 was moderate social support and the score of 3–8 poor social support [14].

## Operational definition

### No depression

Patient Health Questionnaire 9 score of 0–4 [12].

### Depression

Patient Health Questionnaire 9 score of 5–27 [12].

### Adherent

Those hypertensive patients who take at least 4 and above consecutive days out of 7 days, which is greater than or equal to 90% adherence to drugs [15].

### Non-adherent

Those hypertensive patients who take less than 4 consecutive days out of 7 days, which is less than 90% adherence to drugs [15].

### Data collection procedure, quality assurance, processing and analysis

Data was collected by structured interviewer questionnaire and chart review. The data was collected by nurses working at medical outpatient departments of selected hospitals. Adequate training and supervision was given to the data collectors and supervisors. Codes were given to the questionnaire. The filled questionnaire was checked for completeness by the data collector and supervisor every day. Computer frequencies and data sorting were used to check for missed variables, outliers or other errors during data entry. Data was entered using Epi Data 4.6 and exported to a statistical package for social sciences (SPSS) version 25. The analysis was done with descriptive statistics by using frequency, percentage and mean. Multicollinearity test was done among independent variables using the correlation coefficient. Bivariate analysis between dependent and independent variables was also performed using binary logistic regression. All explanatory variables which have an association in bivariate regression analysis with a p-value less than or equals to 0.25 were entered into a multivariable regression model. Hosmer and Lemeshow test was checked for model goodness of fit. In multivariable regression P-value of less than or equal to 0.05 was taken as a significant association.

## Results

### Socio-demographic characteristics

A total of 311 respondents were participated with a response rate of 100. The mean age of the participants was 58.85 ± 10.75. About half of the respondents were females. Regarding social support 53(17%) had poor social support (Table 1).

**Table 1:** Socio-demographic characteristics hypertensive patients on follow–up in South Gondar zone hospitals, Northwest Ethiopia, 2023

Variables	Category	Frequency (N)	Percent (%)
Sex	Male	157	50.5
	Female	154	49.5
Age	18–39	65	20.9
	40–60	125	40.2
	> 60	121	38.9
Marital status	Married	194	62.4
	Single	70	22.5
	Separated	47	15.1
Residence	Rural	175	56.3
	Urban	136	43.7
Educational status	Uneducated	170	54.7
	Educated	141	45.3
Social support	Poor	53	17
	Moderate	160	51.4
	Strong	98	31.5
Monthly income	< 1000	88	28.3
	1000–3559	163	52.4
	3560–10799	37	11.9
	> 10,799	23	7.4

### Behavioral factors

About 23 (7.4%) of the respondents were khat chewer. Nearly 60% of the respondents had irregular physical exercise habits (Table 2).

**Table 2:** Behavioral characteristics of hypertensive patients on follow–up in South Gondar zone hospitals, Northwest Ethiopia, 2023

Variables	Category	Frequency (N)	Percent (%)
Cigarette smoking	Yes	45	14.5
	No	266	85.5
Alcohol drinking	Yes	10	3.2
	No	301	96.8
Khat chewing	Yes	23	7.4
	No	288	92.6
Physical exercise	Irregular	182	58.5
	Regular	129	41.5
Dietary regimen	≤ 2 serving	64	20.6
	> 2 servings	247	79.4

### Disease and medication related factors

More than 60% of the respondents had co-morbid illness. Majority of the participants were not adhered to their medication (Table 3).

**Table 3:** Disease and medication related characteristics of hypertensive patients on follow–up in South Gondar zone hospitals, Northwest Ethiopia, 2023

Variables	Category	Frequency (N)	Percent (%)
Co-morbidity	Yes	194	62.4
	No	117	37.6
Complications	Yes	42	13.5
	No	269	86.5
Medication adherence	Non-adherent	244	78.5
	Adherent	67	21.5
Depression history	Yes	78	25.1
	No	233	74.9
Hypertension control	Not controlled	230	74
	Controlled	81	26
Duration of hypertension	< 5 years	123	39.5
	5–10 years	119	38.3
	> 10 years	69	22.2

### Magnitude of depression

**Fig. 1:**
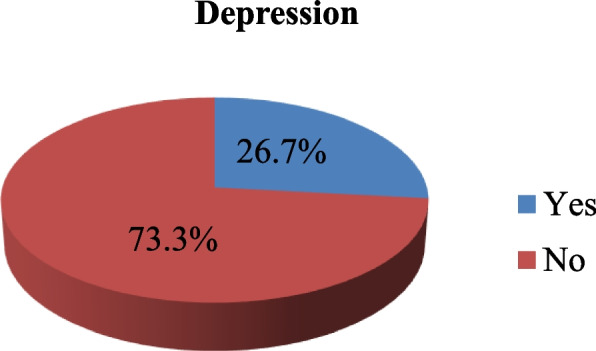
Magnitude of depression among hypertensive patients on follow–up in South Gondar zone hospitals, Northwest Ethiopia, 2023

About 26.7% of hypertensive patients had depression (Fig. 1).

### Factors associated with depression

From the total of eighteen independent variables; thirteen were associated in bivairate regression. Finally, nine independents variables were associated with depression in multivariable regression analysis. Those hypertensive patients who had poor social support were 6.76 times more likely to have depression compared to those patients with strong social support. Hypertensive patients who had co-morbid illness were 2.36 times more likely to develop depression compared to patients without co-morbidity (Table 4).

**Table 4:** Factors associated with depression among hypertensive patients on follow–up in South Gondar zone hospitals, Northwest Ethiopia, 2023

Variables	Category	Depression		COR	P value	AOR	*P* value
		**Yes**	**No**				
Sex	Male	29	128	**1**			
	Female	54	100	2.38(1.42,4.02)	0.001	**2.69(1.23,5.89)**	**0.013**
Age	18–39 years	13	52	**1**			
	40–60 years	23	102	0.9(0.42,1.92)	0.790	2.13(0.74, 6.13)	0.160
	> 60 years	47	74	2.54(1.25,5.16)	0.010	**5.89(2.09,16.61)**	**0.001**
Marital status	Married	42	152	**1**			
	Single	24	46	1.89(1.04,3.44)	0.038	1.57(0.67, 3.71)	0.302
	Separated	17	30	2.05(1.03,4.07)	0.040	2(0.65, 6.14)	0.225
Residence	Rural	57	118	2.04(1.20,3.48)	0.008	1.13(0.46, 2.80)	0.785
	Urban	26	110	**1**			
Educational status	Uneducated	59	111	2.59(1.51,4.45)	0.001	**3.70(1.58,8.70)**	**0.003**
	Educated	24	117	**1**			
Social support	Poor	35	18	7.13(3.39,15.03)	0.000	**6.76(2.54,17.96)**	**0.000**
	Moderate	27	133	0.74(0.39,1.41)	0.363	0.77(0.33, 1.78)	0.542
	Strong	21	77	**1**			
Co-morbidity	Yes	58	136	1.57(0.92,2.69)	0.101	**2.36(1.07,5.18)**	**0.033**
	No	25	92	**1**			
Complication	Yes	22	20	3.75(1.92,7.33)	0.000	**5.95(1.91,18.59)**	**0.002**
	No	61	208	**1**			
Khat chewing	Yes	10	13	2.27(0.95,5.39)	0.064	2.44(0.67, 8.90)	0.176
	No	73	215	**1**			
Medication adherence	Non adherent	72	172	2.13(1.06,4.30)	0.035	1.99(0.69, 572)	0.201
	Adherent	11	56	**1**			
Dietary regimen	≤ 2 serving	27	37	2.49(1.39, 4.44)	0.002	2.51(1.10,5.97)	**0.038**
	> serving	56	191	**1**			
Duration of hypertension	< 5 years	22	101	**1**			
	5–10 years	33	86	1.76(0.96, 3.25)	0.069	1.54(0.65, 3.64)	0.326
	> 10 years	28	41	3.14(1.61, 6.10)	0.001	3.74(1.47, 9.49)	**0.006**
Controls of Hypertension	Not controlled	69	161	2.05(1.08(3.90)	0.028	3.2(1.31, 7.83)	**0.011**
	Controlled	14	67	**1**			

*AOR* Adjusted Odd Ratio, *COR* Crude Odd Ratio, Hosmer and Lemeshow goodness of fit test = 0.671

## Discussion

The main purpose of this study was to assess the magnitude of depression and its associated factors among adult hypertensive patients attending follow-up in south Gondar zone public hospitals, Ethiopia, 2023.

In this study the magnitude of depression was 26.7% [(95%CI (21.9–31.8)]. The finding is lower than the studies done in Afghanistan 58.1% [16], Kanyakumari, India 41% [17], Ghana 41.7% [18], Hadiya zone 37.2% [19], Addis Ababa, Ethiopia 37.8% [20]. This discrepancy might be due to the fact that overcrowding is danger to societal mental health of densely populated and its main adverse effect is depression [21]. Also it can be due to the differences in socio-demographic factors, norms and culture of the communities about the nature of the disease might bring this. But the finding is inline with the studies done in China 26.8% [22], Malaysia, 32% [9], Nigeria 26.6% [18] and Hawassa, Ethiopia was 24.19% [12].

This study confirmed that being elders were more likely to have depression. This finding is similar with the studies done in Afghanistan [16] and Saudi Arabia [1]. The reason found from these studies argued that when age increasing the chance of getting depression was also increased.

Being females were more likely to develop depression. This finding was supported by the Studies done in Indian, Saudi Arabia, Nepal, and Ethiopia [10, 12, 20, 23, 24]. The reason for higher level of depression in females might be result from sex hormonal changes, influences from social norms and gender issues, and parental restrictions toward their daughters as opposed to their sons. These factors can affect daughters' sense of self-control and self-esteem and make them more susceptible to depression. Domestic and sexual abuse might be also factors in the greater risk among females [25].

In this study, uneducated hypertensive participants were more prone to depression compared to educate clients. This finding was similar with the study done in Saudi Arabia, Indian and Ethiopia [1, 10, 12]. The reason might be those educated patients could have better information about the nature of the disease.

Our finding also confirmed that hypertensive patients who had poor social support were more likely to develop depression compared to those who had strong social support. This finding was similar with a study done in Hawassa, Ethiopia [12]. The reason might be these hypertensive patients who have poor social support could be felt loneliness and experienced stressful life which leads to the developments of harmful behaviors like alcohol drinking and khat chewing.

Those hypertensive patients who could not control their hypertension were more likely to develop depression. This finding was consistent with studies done in Saudi Arabia and Ethiopia [1, 12]. This is because of controlling hypertension means the patients can control or free from emotion which might decrease the chance of having depression.

This study revealed that the presence of co-morbid illnesses and complication along with hypertension were significantly attacked by depression. Our finding was similar with the studies done in an Afghanistan [16],India [17], Hadiya zone, Ethiopia [19]. That could be explained by the despair and ongoing complex that co-morbid illnesses bring about [26].

In this study, we found that longer duration of hypertension were more likely to have depression. This was similar with the studies done in Saudi Arabia [1], Pakistan [17], and Ethiopia [12]. The possible explanation is as the patients live longer with the same disease, the less hope they have in a full recovery [1].

In this study we have confirmed that those hypertensive patients who accessed less than two dietary servings were more likely to develop depression. The reason is due to the fact that getting dietary diversity essential for good health. Eat a variety from each of the five food groups (vegetables, fruits, grains, lean meat/poultry and fats) daily in the recommended amounts [27]. We have included participants from multi-center study setting and using the validated tool can contribute to the strength of the study. The limitation of this study is as we employed cross-sectional study design; it did not show the cause effect relationship between the outcome and explanatory variables. Additionally, because of some associated variables are also risk factors for depression alone; this study did not show the specific impact of these variables on depression among hypertensive patients.

## Conclusion

The magnitude of depression was found to be high. This indicated that depression is the common co-morbid illness among hypertensive patients. Health care professionals should consider and diagnose co-morbid illness like depression among hypertensive patients on follow-up. Depression should be assessed routinely among hypertensive patients at each visiting during follow-up. It is also better to give special emphasis for highly vulnerable groups like female, elders, uneducated, having poor social support, those who developed co-morbid illnesses and complications, uncontrolled hypertension, patients who only accessed less than two dietary servings and those clients living with hypertension for long durations.

## Data Availability

Data is provided within the manuscript files and the datasets used or analyzed during the current study are available from the corresponding author on reasonable request.
